# Understanding contagion dynamics through microscopic processes in active Brownian particles

**DOI:** 10.1038/s41598-020-77860-y

**Published:** 2020-11-30

**Authors:** Ariel Norambuena, Felipe J. Valencia, Francisca Guzmán-Lastra

**Affiliations:** 1 Centro de Investigación DAiTA Lab, Facultad de Estudios Interdisciplinarios, Universidad Mayor, Santiago, Chile; 2 Centro para el Desarrollo de la Nanociencia y la Nanotecnología, CEDENNA, Avda. Ecuador 3493, 9170124 Santiago, Chile; 3 Escuela de Data Science, Facultad de Estudios Interdisciplinarios, Universidad Mayor, Santiago, Chile

**Keywords:** Biological physics, Applied physics

## Abstract

Together with the universally recognized SIR model, several approaches have been employed to understand the contagion dynamics of interacting particles. Here, Active Brownian particles (ABP) are introduced to model the contagion dynamics of living agents that perform a horizontal transmission of an infectious disease in space and time. By performing an ensemble average description of the ABP simulations, we statistically describe susceptible, infected, and recovered groups in terms of particle densities, activity, contagious rates, and random recovery times. Our results show that ABP reproduces the time dependence observed in traditional compartmental models such as the Susceptible-Infected-Recovery (SIR) models and allows us to explore the critical densities and the contagious radius that facilitates the virus spread. Furthermore, we derive a first-principles analytical expression for the contagion rate in terms of microscopic parameters, without considering free parameters as the classical SIR-based models. This approach offers a novel alternative to incorporate microscopic processes into analyzing SIR-based models with applications in a wide range of biological systems.

## Introduction

Mathematical models and computational calculations provide powerful scientific tools to understand and predict future scenarios associated with viral propagation dynamics. Historically, infectious diseases have been modeled using SIR-based models^1^, which include phenomenological rates describing contagion, recuperation, death, or quarantine. Nevertheless, a more realistic model must consider the mobility of infectious particles and particle density within its environment. In this direction, self-propelled particles^2,3^, the random motion of non-interacting particles^4,5^, cellular automaton^6,7^, dynamical density functional theory approach^8^, and reaction–diffusion models^9,10^ have been proposed to introduce the spatial motion of infectious particles. As a matter of universality, active matter models are intuitive and are extensively used to describe a wide range of biological processes ranging from bacteria motion to animal movement^11^. Thus, as active matter lies at the core of almost all biological processes, it emerges as an excellent and non-explored candidate to describe the contagion dynamics between moving agents.

Active matter (AM) affects the organization and collective behavior of living organisms on all length scales, ranging from cytoskeleton on the nanoscale through cheeps on the mesoscale^12–14^. Since the work of self-driven particles of Viscek et al.^15^, the modeling of active agents has been possible following a series of rules for particle interactions, such as alignment, polarization, repulsion, and quorum-sensing^13,16^. These interactions often give rise to the understanding of unexpected phenomena such as collective motion, turbulence, giant fluctuations, rectification, and self-organization^16–20^, and at the same time, they reproduce what we observe in nature. At the micro-scale, agents can be modeled as active Brownian particles (ABP), where ABP can take up energy from the environment to store it in an internal depot and convert it internal energy into kinetic energy and motion^21^. Therefore, thermal fluctuations in these systems are dominant for active^17,22,23^ and non-active^24–27^ particles. Furthermore, ABP has been tested reproducing either biological processes or artificial ones in several studies where it seems that activity and short-range interactions are enough to understand particle-particle and particle-surface interactions^21,28^.

At the mesoscale, inertia and viscous forces are balanced; however, this regime has been less explored^16,29^. Although living systems in this length scale are plenty, such as marine and aerial groups of animals, their modeling is less unified since their dynamics depend on the fluid media where they move and because particle interactions get more specific in function on the target problem^18,29^. For instance, human mobility has been modeled using self-propelled models with a gravitational term that describes the ambient information^30,31^. Other approaches to the dynamics of infectious diseases in humans^32^ have also been explored using a non-linear wave approach by means of reaction–diffusion equations to model the effect of random motion in the SIR dynamics. In our case, those conditions can be reproduced in the limit where the activity is zero, and the particles perform only Brownian motion. In this, aspect AM offers the possibility to simulate the way that the walkers interact, to obtain the typically S(t), I(t), and R(t) curves. Besides, AM introduce an empirical potential modeling the repulsion between particles and an additional rotational diffusion term that controls the medium’s exploration. This is crucially different from the non-linear wave equations, leading to AM simulations to model complex effects such as clusterization formation or bimodal phase separation, which usually are not captured by the typical non-linear wave approaches.

Here, we explore infection propagation through active vectors that carry an internal state using an AM based model with underlying microscopic processes. The infection occurs through horizontal transmission when a susceptible agent comes into contact with an infected agent such as viruses propagating in salmon hatcheries, honeycombs^33,34^, or in mesoscale organisms such as cats with influenza or humans carrying flu^2,3,35^.

**Figure 1:**
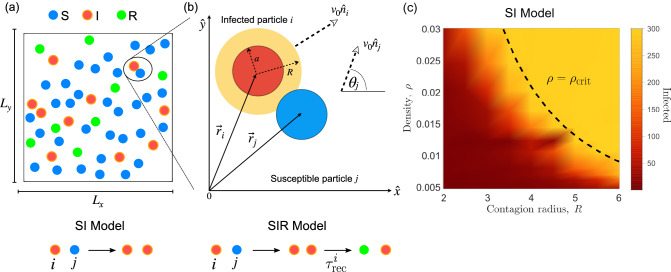
Schematic representation of the AM model based on ABP. (**a**) Sketch of the simulation box: *N* moving particles in a rectangular box of size $L_x \times L_y$ with periodic boundary conditions. In all simulations, we randomly set the initial positions $\vec r_i$ and orientations ${{\hat{n}}}_i$ for all particles *i* and we consider $I(0) = 1$. (**b**) Particle infection: pair interactions between particle *i* and *j* at a distant $|\vec {r}_i-\vec {r}_j| \le R$, where *R* is the contagion radius. Infected particle *i* is moving with velocity $v_0{\hat{n}}_i$ and given position $\vec r_i$ and interact through the contagion radius *R* with particle *j* which is moving with velocity $v_0{\hat{n}}_j$ and position $\vec r_j$. For the SI model we only consider two states: susceptible *S*(*t*) (blue) and infected *I*(*t*) (red) such that $S(t) + I(t) = N$. In the SIR model, we introduced the recovered group *R*(*t*) (green) such that after a random recovery time $\tau _{\mathrm{rec}}^{i}$ infected particle *i* becomes recovered. (**c**) Phase diagram for the SI model showing the number of infected particles as a function of the contagion radius *R* and the particle density $\rho = N/A$. The dashed black line represent the critical density $\rho _{\mathrm{crit}} = 1/(\pi R^2)$. For the simulation we consider $N= 300$, $v_0 = 1$, $L_x = L_y$, and $I(0)=1$.

## Results

### Active Brownian particles and SI model

Let us consider *N* moving particles in a rectangular box with area *A* and periodic boundary conditions, as shown in Fig. 1a. We model the ABP dynamics by considering both Weeks–Chandler–Andersen (WCA) potential (5) and rotational diffusion according to the following set of Langevin equations

1 $$\begin{aligned} \dot{\vec {r}}_i = -\sum _{j \ne i} \vec {F}_{i j} + v_0 {\hat{n}}_i, \quad {\dot{\theta }}_{i}=\xi _{i}^{\theta }, \quad i =1,...,N \end{aligned}$$

where $\vec {r}_i = (x_i,y_i)$ is the instantaneous position, $\vec {F}_{i j} = -\nabla U_{ij}$ is the force derived from the WCA potential, ${\hat{n}}_i=(\cos \theta _i,\sin \theta _i)$ is the director vector, and $\theta _i = \tan ^{-1}(y_i/x_i)$ is the orientation. Due to particle rotational diffusion, each angle $\theta _i$ change randomly according to the Wiener process, where $\left\langle \xi _{i}^{\theta }(t)\right\rangle =0$ and $\left\langle \xi _{i}^{\theta }(t) \xi _{i}^{\theta }(0)\right\rangle =2 D_{R} \delta (t)$. Here, $D_R$ is assumed as a constant parameter that takes account particle’s exploration of the medium. Then, active agents are allowed to move persistently in their given direction, with a persistence length $l_p=v_0/2D_R$, until a random reorientation takes place^36^. Therefore when $D_R$ is small active agents can be considered as persistent explorers, which is true for several organisms in nature^12^.

First, we consider a simple SI model where infected *I*(*t*) and susceptible *S*(*t*) satisfy $I(t) + S(t) = N$. A contagious event occurs when a susceptible particle *i* is in contact with an infected particle *j* at a distant $|\vec {r}_i-\vec {r}_j| \le R$, where *R* is the contagion radius, as shown in Fig. 1b. For a set of parameters $(N,L_x,L_y,R,v_0)$ we run $N_{\mathrm{sim}}$ simulations to compute the ensemble average curves $I(t) = \sum _{i=1}^{N_{\mathrm{sim}}} I_i(t)/N$ and $S(t) = \sum _{i=1}^{N_{\mathrm{sim}}} S_i(t)/N$. In Fig. 1c, we show a phase diagram for the number of infected as a function of the contagion rate and the particle density for $N = 300$. As expected, in the region of high density and large contagion radius, the infected group saturates reaching its maximum value. More importantly, we observe the existence of a critical density $\rho _{\mathrm{c}} = 1/(\pi R^2)$ (black dashed line) above which the particles are immediately infected.

It is worth to notice that in several situations, living organisms do not move randomly. This is especially true for human^13,37^, or even in animals^38^; However, there are situations where the persistence of the movement is altered by ambient noise, for example, humans moving inside a supermarket or in a shopping mall, on those scenarios the movement is altered to avoid clashes between individuals as they also performed some random explorations of the medium. Although humans might not be considered as random particles, in a first approximation, humans and animals in all length scales can be considered as active random particles^12,14^ such as fishes, mosquitos, bees, algae, and bacteria, among others^15^, in this case, active matter models can correctly describe agents interactions and collective motion^39^.

### Microscopic expression contagion rate

Using a mean-free-path analysis (see Methods 4.2 for further details), we obtain the following analytical expression for the contagion rate *r*:

2 $$\begin{aligned} r = \frac{\sqrt{8} \rho R v_0 }{1-\rho /\rho _{\mathrm{crit}}}, \quad \quad 0 \le \rho \le \rho _{\mathrm{crit}}. \end{aligned}$$

In the low-density regime, $\rho \ll \rho _{\mathrm{crit}}$, we obtain a linear scaling $r \approx \sqrt{8} \rho R v_0$. Also, our model predicts a singularity at $\rho = \rho _{\mathrm{crit}}$ for which $r \rightarrow \infty$. As a result, all particles are instantaneously infected. One critical observation is the dimensional-dependent nature of the contagion rate in our model. For instance, for *N* moving particles in a volume *V*, the mean-free-path analysis predicts a three-dimensional contagion rate $r^{3\mathrm D} = \pi \rho ^{3\mathrm D}R^2\langle v_{\mathrm{rel}}\rangle /(1-\rho ^{3\mathrm D}/\rho ^{3\mathrm D}_{\mathrm{crit}})$, where $\rho ^{3\mathrm D} = N/V$, $\rho ^{3\mathrm D}_{\mathrm{crit}} = 1/(4/3\pi R^3)$, and $\langle v_{\mathrm{rel}}\rangle$ is the average relative velocity between particles. Therefore, our active matter model predicts that distancing between infected particles is more critical in a three-dimensional system since $r^{3\mathrm D} \propto R^2$. The latter can be crucial in biological systems where a 3D movement is present during the contagion dynamics^12,29^.

**Figure 2:**
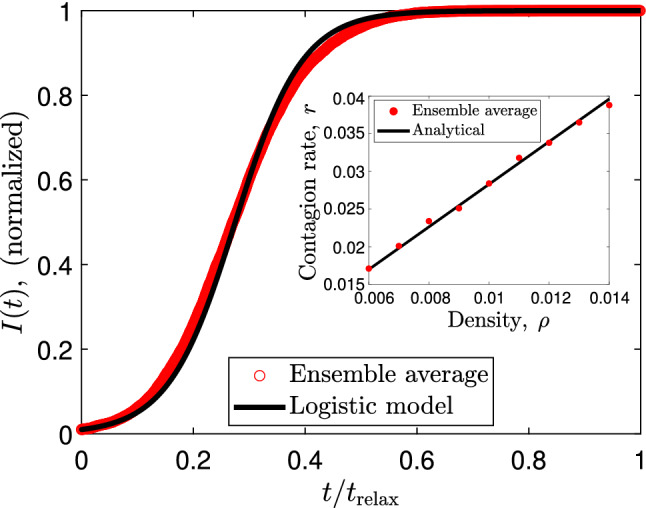
Time evolution of the infected group for the SI model. The red circles are numerical simulations of the Langevin equations after calculating the ensemble average. The solid black line is the solution of Eq. (3). For the simulation we use $N = 100$, $R=1$, $L_x = L_y = 100$, and $v_0 =1$. Here, $t_{\mathrm{relax}}$ is the relaxation time required to find the stationary state of the system. The inset plot show the contagion rate as a function of the particle density, where we compare the analytical expression derived in (2) (solid line) with our simulation (red circles). For the simulation we use $N=100$, $N_{\mathrm{sim}} = 100$ $R=1$, $L_x = L_y$, and $v_0 =1$.

Our model has minimal but fundamental mechanisms to study clustering formation (CF)^39^ or two-phase separation (TPS)^40^ while varying the particle activity (velocity) or density^23,41^. In the scenario of virus propagation, a TPS could be relevant since CF might be included in the contagion dynamics by considering density gradients or hot spots in dilute or dense systems. The latter can be used to simulate quarantines in groups or on their city hall while some rangers continue moving in the space between clusters. In this case, we expect that the density-dependent contagion rates *r* and $r^{3\mathrm D}$ would be useful for novel mechanisms of infection that are not described by standard epidemic models^41^.

**Figure 3:**
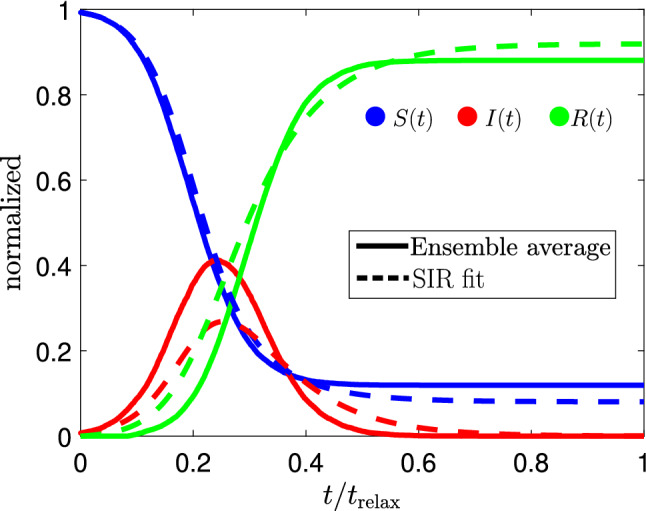
Comparison between our SIR model and the best fit obtained by optimizing the parameters $\alpha$ and $\beta$. For the simulation we consider one initial infected particle, $I(0) =1$ and a random recovery time $\tau _{\mathrm{rec}}^{i} \in [30,50]$. For the numerical calculations we use $N=150$, $R=1$,$L_x = L_y = 100$, and $v_0 = 1$.

Now, we shall establish the connection between our microscopic contagion rate given in Eq. (2) and the characteristic epidemic curve for the SI model. At each discrete time $t_n = n\Delta t$ ($n \in \mathbb {N}$ and $\Delta t >0$), the number of infected varies according to the Markovian model $I_{n+1} = I_n + p_n S_n$, where $p_n = (r \Delta t)(I_n/N)$ and $S_n = N - I_n$ are the contagion probability and number of susceptible at time $t_n$, respectively. As a consequence, in the continuum limit, the curve *I*(*t*) evolves according to ($\Delta t \rightarrow 0$):

3 $$\begin{aligned} {\dot{I}} = rI\left( 1-\frac{I}{N} \right) , \quad S(t) = N-I(t). \end{aligned}$$

The above equations can be written as ${\dot{S}} = -r I S /N$ and ${\dot{I}}= r I S /N$, which is the standard SI model. The logistic function $I(t) = I(0)Ne^{r t}/[(N-I(0))+I(0)e^{rt}]$ gives the analytic solution of (3). To support our previous observations, in Fig. 2, we plot a comparison between the infected curve *I*(*t*) obtained from the ensemble average procedure and the logistic model given above. Here, we consider a system with $N=100$ particles in a square box with lengths $L_x = L_y =100$, contagion radius $R=1$, and particle velocity $v_0 = 1$. We observe a good agreement between the theory and simulations, revealing that one initial contagion grows logistically if the recovered group is neglected. However, a small asymmetry of the analytical logistic model is observed in Fig. 2. One suggestive approach is to fit the ensemble average with the generalized logistic model or Richard’s model given by ${\dot{I}} = r I^p[1-(I/N)^q]$ ($0 \le p \le 1$) which has been used in COVID-19 pandemic curves^42^. This could be useful for biological systems showing logistic-like behaviors with more complicated microscopic dynamics.

Furthermore, in the inset of Fig. 2, we compare the microscopic expression for the contagion rate defined in Eq. (2) and the predicted rate obtained in our simulations. We recover the predicted linear dependence of the contagion rate in terms of the particle density, which validates our microscopic model.

### Active Brownian particles and SIR model

Now, we include the recovered group *R*(*t*) into the dynamics. In such a case, the total number of particles satisfy $S(t) + I(t) + R(t) = N$. First, we assume that the recovered group cannot be infected again, that is, particles gain immunity. Second, we neglect deaths since we are interested in the propagation itself. Third, we introduce a random recovery time $\tau _{\mathrm{rec}}^{i}$ for each particle ($i=1,...,N$) such that $\tau _{\mathrm{rec}}^{i} \in [\tau _{\mathrm{min}}, \tau _{\mathrm{max}}]$. Here, $\tau _{\mathrm{min}}$ and $\tau _{\mathrm{max}}$ are the minimum and maximum recovery in our simulations, respectively. In what follows, we use a uniformly distributed random number to generate the individual recovery times. This particular choice of the distribution impacts on the symmetry properties of the infected curve; thus, other probability distributions can be used to simulate a different scenario. Also, other relevant times, such as the incubation time, can be incorporated into the dynamics, which originates a delayed dynamics for the infected curve, as discussed in Ref.^43^. For simplicity, we only consider the effect of the recovery time on the dynamics.

We compare our simulations with the conventional SIR model, which is described by the set of differential equations ${\dot{S}} = -\alpha IS$, ${\dot{I}} = \alpha I S - \beta I$, ${\dot{R}} = \beta I$, where $\alpha$ and $\beta$ are the infection and recovery rates, respectively^1^. We can find the optimal parameters $\alpha$ and $\beta$ that improves the fit between the SIR model and our simulations. In Fig. 3, we observe a comparison between our simulations (ensemble average) and the SIR fit (dashed lines). In general, we numerically corroborate that our model cannot be fully explained in terms of the standard SIR model. In particular, the SIR model predicts an asymmetry curve for *I*(*t*), and the stationary states differ with our calculations. Our simulations show an asymmetric curve for the infected group, which has been previously observed in Ref.^44^.

**Figure 4:**
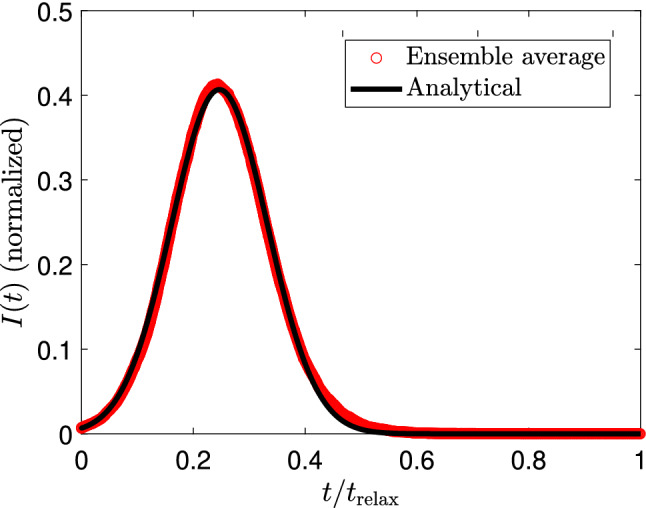
Infected curve and analytical Gaussian prediction for the SIR model. For the numerical calculations we use $N=150$, $R=1$,$L_x = L_y = 100$, $v_0 = 1$, $I(0) =1$, and $\tau _{\mathrm{rec}}^{i} \in [30,50]$.

Using the relations $\alpha = r/N$ (*r* given in Eq. (2)) and $\beta = 1/T_{\mathrm{prom}}$ ($T_{\mathrm{prom}} = (\tau _{\mathrm{min}}+\tau _{\mathrm{max}})/2$) into the equation ${\dot{I}} = r IS /N - I/T_{\mathrm{prom}}$ we can find *I*(*t*). Noticing that relevant contributions to the product *IS* comes from the region where *S*(*t*) has a linear dependence, we use $S(t) = S_0 - m t$ into the dynamics of *I*(*t*), and we found the following Gaussian curve:

4 $$\begin{aligned} I(t) = I(0)e^{\left( {t_0 \over \sqrt{2}\sigma }\right) ^2}e^{-\left( {t-t_0 \over \sqrt{2}\sigma }\right) ^2}, \end{aligned}$$

where $t_0 = (r S_0 - N/T_{\mathrm{prom}})/(r m)$ is the position of the peak and $\sigma = [N/(rm)]^{1/2}$ is the width of the Gaussian curve. In Fig. 4, we observe the good agreement between our simulations and the Gaussian model given in (4). On the one side, the maximum number of infected is estimated as $I_{\mathrm{max}} \approx I(0)\text{ exp }[(t_0/(\sqrt{2}\sigma ))^2]$, and thus the ratio $t_0/\sigma$ is critical. In the low-density regime, we obtain $I_{\mathrm{max}} \propto \text{ exp }[R v_0/A]$ illustrating that the contagion radius, available area, and velocity of particles strongly impact the maximum number of infected during the dynamics. On the other hand, the scaling $\sigma \propto [R v_0/A]^{-1/2}$, tell us that any reduction of the maximum number of infected implies a flattened effect on the curve *I*(*t*), as expected in the standard SIR model. Further improvements or extensions of the current model can be performed by considering the incubation time, different particle velocities, time-dependent densities to model lock-down, or by including particle interactions modeled with microscopic pedestrian models^45^.

## Conclusions

Active Matter (AM) simulations show that active Brownian particles that exchange an internal state can successfully reproduce the universally accepted SIR contagious curves, for horizontal disease transmission, by introducing the effects of contagious radii, particle velocity, and particles density. Theoretically, the SIR model assumes several empirical parameters in order to describe the contagious dynamics. Here, we introduce a first-principle analytical expression that successfully reproduces our simulations in terms of controllable microscopic parameters. Besides, our expression qualitatively recovers the SIR based models with good agreement with numerical simulations. Furthermore, we find an important dependence on the particle density and contagious radius in two and three dimensions, which opens a new forecast parameter in viruses propagation inside a specific population.

Although our study focuses mainly on particle density and contagious rates, we expect that our model can be improved by including complex interactions such as quorum-sensing to describe viruses propagation in birds or schools of fish or by adding external forces to describe human will^3,30,31^. Nevertheless, how these new interactions alter and couple the dynamics with compartmental models are exciting new questions that our work opens to the active matter community.

## Methods

### Brownian Dynamics Simulations in the overdamped limit

We performed Brownian dynamics simulations for $N=300$ disk particles of radius $a=0.5$ [m] bounded in a rectangular box with periodic boundary conditions. Particles are settled initially at random positions and orientation following a uniform distribution. Particles move according to Langevin equations (1) with self-propelled velocity $v_0=1$ [m/s] and rotational diffusion given by $D_R=1$ [rad$^2$/s], where we set a new position and orientation for each particle using the Euler iteration method with a time step $dt=10^{-3}$. Since the particle dynamics is non-deterministic and particle encounters determine the contagious rate, we performed 100 different numerical simulations starting with a different random configuration. Particles perform pair-hard core interactions via the Weeks–Chandler–Andersen (WCA) potential,

5 $$\begin{aligned} U_{ij} = \left\{ \begin{array}{cc} \displaystyle {4 \varepsilon \left[ \left( \frac{r_0}{r_{i j}}\right) ^{12}-\left( \frac{r_0}{r_{ij}}\right) ^{6}\right] } &{} r_{i j} \le r_{m}\\ 0 &{} \text{ otherwise } \end{array} \right. \end{aligned}$$

Here, $\varepsilon$ is the interaction potential constant, $r_m$ locates the potential minimum, which is equal to the limit distance between particles $r_0=2a$. Although this interaction avoids particles overlapping its principal consequence, the particle trajectory deviations imitate living organisms’ encounters. Particles also transmit the infection through an instantaneous pair-interaction, which sets a new length parameter on the problem, the contagious radii *R*. Then if the distance between a susceptible particle and an infected particle is less than *R*, the susceptible particle is labeled as infected. We vary the contagious radii from $R=a,\ldots ,6$, in steps of $\Delta R=0.5$, and the box length $L=100,\ldots ,300$ in increments of $\Delta L=10$^8,46^.

### Microscopic contagion rate

The microscopic contagion rate can be derived using the concept of mean free path $\lambda$, extensively used in the kinetic theory of gases and also used in Ref.^47^. In this context, $\lambda$ represent the mean distance traveled by ABP between successive encounters with other particle at a distance $d_{ij} = R$. In an active media with *N* moving particles $\lambda = \sqrt{\langle |\vec {v}_{\mathrm{rel}}|^2\rangle } \tau _c$, with $\vec {v}_{\mathrm{rel}}$ and $\tau _c$ being the relative velocity between particles and the mean contagion time, respectively. Here, $\langle ...\rangle$ denote the particle average. Thus, we estimate the contagion rate through the relation $r = \tau _c^{-1}$. Encounters between ABP’s depends on the relative velocity $\vec {v}_{\mathrm{rel}} = \vec {v}_i - \vec {v}_j$ ($i\ne j$), from which it follow that $\langle |\vec {v}_{\mathrm{rel}}^{\; ij}|^{2}\rangle = \langle v_i^2\rangle + \langle v_i^2\rangle - 2 \langle \vec {v}_i \cdot \vec {v}_j \rangle$. First, we assume uncorrelated particle’s velocities yielding $\langle \vec {v}_i \cdot \vec {v}_j \rangle = 0$. Second, if the WCA potential does not drastically change the speed $v_0$, we approximately obtain that $\langle |\vec {v}_{\mathrm{rel}}^{\; ij}|^{2}\rangle \approx 2 v_0^2$ since $\langle v_i^2\rangle \approx v_0^2$. By considering the total area swept for *N* particles in a time interval $\tau _c$ as $A_{\mathrm{sw}} = N(2R\lambda + \pi R^2)$, we define the maximum contagion probability $p_{\mathrm{c}} = A_{\mathrm{sw}}/A = 1$, and using the relation $\lambda = \sqrt{2}v_0 \tau _c$, we recover the analytical expression of the contagion rate given in (2).
