# Author Correction: Understanding contagion dynamics through microscopic processes in active Brownian particles

**DOI:** 10.1038/s41598-022-08006-5

**Published:** 2022-03-04

**Authors:** Ariel Norambuena, Felipe J. Valencia, Francisca Guzmán‑Lastra

**Affiliations:** 1 Centro de Investigación DAiTA Lab, Facultad de Estudios Interdisciplinarios, Universidad Mayor, Santiago, Chile; 2 Centro para el Desarrollo de la Nanociencia y la Nanotecnología, CEDENNA, Avda. Ecuador 3493, 9170124 Santiago, Chile; 3 Escuela de Data Science, Facultad de Estudios Interdisciplinarios, Universidad Mayor, Santiago, Chile

Correction to: *Scientific Reports* https://doi.org/10.1038/s41598-020-77860-y, published online 30 November 2020

The original version of this Article contained an error in Reference 4, which was incorrectly given as:

Rodriguez, J. P., Ghanbarnejad, F. & Eguíluz, V. M. Particle velocity controls phase transitions in contagion dynamics. *Sci. Rep.* **9**, 1–9 (2019).

The correct reference is listed below:

Rodríguez, J. P., Ghanbarnejad, F. & Eguíluz, V. M. Particle velocity controls phase transitions in contagion dynamics. *Sci. Rep.* **9**, 6463 (2019).

The original Article has been corrected.

